# Seaweed Secondary Metabolites with Beneficial Health Effects: An Overview of Successes in In Vivo Studies and Clinical Trials

**DOI:** 10.3390/md18010008

**Published:** 2019-12-20

**Authors:** Gonçalo P. Rosa, Wilson R. Tavares, Pedro M. C. Sousa, Aida K. Pagès, Ana M. L. Seca, Diana C. G. A. Pinto

**Affiliations:** 1 cE3c—Centre for Ecology, Evolution and Environmental Changes/Azorean Biodiversity Group & University of Azores, Rua Mãe de Deus, 9501-801 Ponta Delgada, Portugal; goncalo.p.rosa@uac.pt; 2 Faculty of Sciences and Technology, University of Azores, 9501-801 Ponta Delgada, Portugal; wrt-94@hotmail.com (W.R.T.); sdoffich@gmail.com (P.M.C.S.); aidakane.1@hotmail.com (A.K.P.); 3 QOPNA & LAQV-REQUIMTE, Department of Chemistry, University of Aveiro, 3810-193 Aveiro, Portugal

**Keywords:** seaweeds, secondary metabolites, in vivo studies, clinical trials, health effects, dieckol, eckol, fucoxanthin, kahalalide F

## Abstract

Macroalgae are increasingly viewed as a source of secondary metabolites with great potential for the development of new drugs. In this development, in vitro studies are only the first step in a long process, while in vivo studies and clinical trials are the most revealing stages of the true potential and limitations that a given metabolite may have as a new drug. This literature review aims to give a critical overview of the secondary metabolites that reveal the most interesting results in these two steps. Phlorotannins show great pharmaceutical potential in in vivo models and, among the several examples, the anti-dyslipidemia activity of dieckol must be highlighted because it was more effective than lovastatin in an in vivo model. The IRLIIVLMPILMA tridecapeptide that exhibits an in vivo level of activity similar to the hypotensive clinical drug captopril should still be stressed, as well as griffithsin which showed such stunning results over a variety of animal models and which will probably move onto clinical trials soon. Regarding clinical trials, studies with pure algal metabolites are scarce, limited to those carried out with kahalalide F and fucoxanthin. The majority of clinical trials currently aim to ascertain the effect of algae consumption, as extracts or fractions, on obesity and diabetes.

## 1. Introduction

In the last few years, macroalgae attracted increasing attention from many industries of diverse branches such as fuel, plastics, cosmetics, pharmaceuticals, and food [1,2]. In fact, the chemical diversity within red (Rhodophyta), green (Chlorophyta), and brown (Phaeophyta) macroalgae offers the possibility of finding a wide variety of primary and secondary metabolites, with interesting properties and applications [1,3,4,5,6,7]. Primary metabolites are directly involved in physiological functions, under normal growth conditions, such as reproduction, while secondary metabolites are mainly excretory products produced under different stress conditions, such as exposure to ultraviolet (UV) radiation, changes in temperature and salinity, or environmental pollutants. Primary algal metabolites are the normal ones, such as proteins, polysaccharides, and lipids, whereas the main secondary metabolites produced in algae tissues are phenolic compounds, halogenated compounds, sterols, terpenes, and small peptides, among other bioactive compounds [8,9,10,11].

Studies focusing on the preparation of macroalgae extracts and their chemical characterization revealed a large range of seaweed compounds with very interesting biological activities including antitumor, anti-inflammatory, antimicrobial, antidiabetic, antivirus, antihypertensive, fat-lowering, and neuroprotective activities [12,13,14,15].

The large volume of studies proving the seaweed compound activities in in vitro systems [16,17,18,19] hints the need for further advancements in the knowledge about macroalgae compound efficiency in living systems (in vivo) and their use in the development of pharmaceuticals. In vitro studies are very relevant and yield very important information, but they only represent the first step of a long process, and the results obtained rarely reveal anything about the effects of a compound in vivo, because the responses observed in vitro can be magnified, diminished, or totally different in a more complex and integrated system. In fact, in vivo studies and clinical trials are those which contribute most to truly understanding the real potential of compounds as future pharmaceuticals.

In this regard, the present work intends to present insight into the results obtained in the last few years regarding secondary metabolites, such as phlorotannins, halogenated compounds, fucoxanthin, and fucosterol isolated from macroalgae, involved in in vivo studies and clinical trials, identifying the research opportunities and knowledge gaps, to valorize these compounds and their natural resources. The intention is not to present an exhaustive survey of all published works, but rather a selection of authors based on the following criteria: in-depth studies involving pure compounds most characteristic from seaweeds, and studies in which the applied dose was less than 100 mg/kg, with a few exceptions justified in the discussion of these studies.

## 2. In Vivo Studies

Several compounds isolated from macroalgae reached the in vivo stage of investigation into their biological effects, which means that researchers recognize their potential and are willing to prove their full pharmacological value. In this regard, the paragraphs below review and discuss the most significant results obtained in these in vivo studies.

### 2.1. Phlorotannins

**Figure 1:**
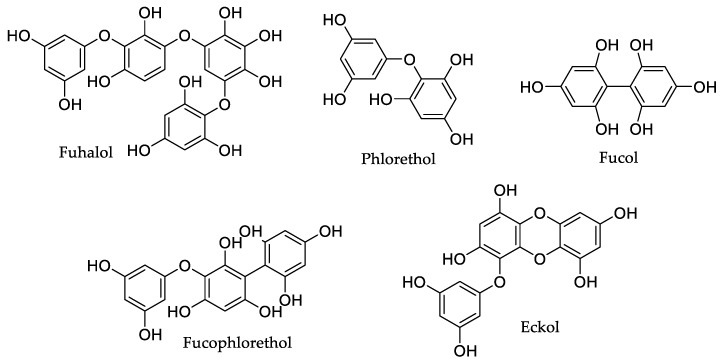
Examples of different subclasses of phlorotannins.

Phlorotannins are a class of inimitable complex polyphenol compounds produced by brown seaweed as secondary metabolites and biosynthesized via the acetate malonate pathway [20,21]. They are basically constituted by phloroglucinol (1,3,5-trihydroxybenzene) base units with different degrees of polymerization. Phlorotannin classification is based on the types of linkages between the phloroglucinol units, and there are four subclasses, namely, phlorotannins with ether linkages (fuhalols and phlorethols), those with phenyl linkages (fucols), those with both ether and phenyl linkages (fucophlorethols), and those with a dibenzodioxin linkage (eckols) [22] (Figure 1).

Phlorotannin presence, either in free form or forming complexes with different components of the cell walls, like alginic acid [23], is essential to the physiological integrity of algae and to numerous important other roles such as chemical defense against bacteria, epiphytes, and hydroids, protection against oxidative damage that occurs in response to interactions with other organisms or the abiotic environment such as UV radiation, and changes in nutrient availability [24,25].

**Figure 2:**
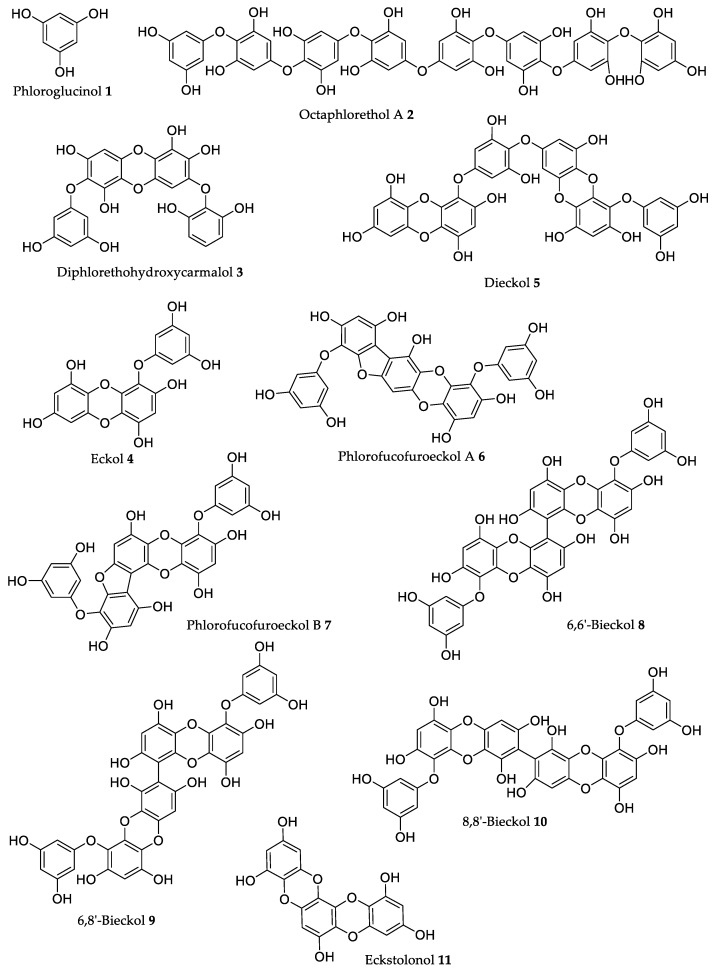
Chemical structures of phlorotannins referred to in Table 1 with relevant in vivo activities.

Due to their important roles in the physiology of brown algae, these compounds attracted a lot of research interest, with many studies addressing their isolation [26,27,28,29]. Moreover, as reviewed by Imbs and Zvyagintseva [30], there were a high number of studies describing the important in vitro activities of phlorotannins including anti-inflammatory, antitumor, and antibacterial activities, among others, which led researchers to advance the study of these compounds, trying to prove their biological activities in vivo. The main results of those studies are summarized in Table 1, and the most relevant aspects are discussed below, while the compounds’ chemical structures are presented in Figure 2. 

#### 2.1.1. Phloroglucinol

Phloroglucinol **1** (Figure 2), the basic unit of phlorotannins, was found to reduce H_2_O_2_-induced toxicity in zebrafish, with the treated group (50 µM of **1**) presenting a survival rate of 90% against only 60% in the control group [34]. The augmented survival rate was correlated with a reduction of H_2_O_2_-induced cell death, lipid peroxidation, and ROS formation. Moreover, this compound accelerates liver regeneration after metronidazole (MNZ)-induced apoptosis at a concentration of 400 µM [34].

The effects of **1** on the blood glucose level and the regulation of glucose synthesis in the liver were also investigated. As shown in Table 1, phloroglucinol **1** (100 mg/kg b.w.) significantly improved glucose tolerance in C57BL/6J male mice whose diet was high in fat and inhibited glucose synthesis in primary mouse hepatocytes [37]. This phlorotannin also exerts efficient cell protection against ionizing radiation and extends the survival of mice exposed to a lethal dose of γ-radiation. Thirty days after exposure, there was a survival rate of 90% in the group treated with 100 mg/kg (b.w) of **1** and 70% in the group treated with 50 mg/kg (b.w.), while, in the control group, only 40% of the mice survived [32]. It was proposed that the protection against γ-radiation is mainly due to the antioxidant effects of **1**, namely, the inhibition of ROS formation, leading to the inhibition of mitogen-activated protein kinase kinase-4 (MKK4/SEK1), c-Jun NH_2_-terminal kinase (JNK), and activator protein-1 (AP-1) cascades [32,69]. Moon et al. [33] found that administration of **1** (20 mg/kg b.w.) could enhance the jejunal crypt survival by 26.4% and decreased the number of apoptotic cells in the jejunal crypts by 32.5% when compared with the untreated irradiated group (Table 1).

Phloroglucinol **1** (100 mg/kg b.w.) protects hairless mice against UV-B-induced photodamage in the skin, by significantly reducing (25%–75%) wrinkle formation, epidermal thickness, and elastic fiber degeneration [38]. The levels of UV-B-induced DNA damage are also decreased by **1** since the topical application of 10 mg/mouse was found to increase the expression levels of xeroderma pigmentosum complementation group C (XPC) and excision repair cross-complementation 1 (ERCC1). These components are essential for the activation of the nucleotide excision repair (NER) pathway, which is the mechanism responsible for DNA repairing [39]. Phloroglucinol **1** also exhibits breast anticancer activity at 25 mg/kg (b.w.), either by decreasing tumor growth or by suppressing the metastatic ability of breast cancer cells that spread to the lungs, contributing in both cases to an increase of survival time in mice (Table 1) [35,36]. Since there is still no suitable therapeutic agent that blocks the progression of breast cancer, these results can be of clinical importance for the treatment of metastatic breast cancer.

#### 2.1.2. Octaphlorethol A

Octaphlorethol A **2**, a rare phlorotannin, decreased oxidative stress induced either by 2,2′-azobis (2-amidinopropane) (AAPH) [42] or by high levels of glucose [41] in zebrafish embryos (Table 1). This phlorotannin is toxic for the embryos at concentrations above 50.4 μM; however, at concentrations lower than 25.2 μM, a strong antioxidant effect was noted without traces of toxicity [42]. These toxicity values against zebrafish are supported by the data obtained by Kim et al. [43], which found that more than 90% of subject embryos survived upon exposure to **2** at concentrations below 25 μM, which was not significantly different from the findings in the control group. Moreover, the same authors reported that this compound significantly inhibited melanin synthesis (27.8%) and tyrosinase activity (32.8%) at a concentration of 25 μM, which is higher than the 15% and 17.3% of inhibition obtained with the reference compound, arbutin, at 500 μM, for melanin synthesis and tyrosinase activity, respectively. These results indicate that **2** has a potential for application in skin-whitening formulations [43].

A dose of 10 mg/kg (b.w.) of **2** led to a reduction of 21.9 mmHg in the systolic blood pressure (SBP) in spontaneously hypertensive rats (SHR), against the 26.3 mmHg reduction obtained using the same dosage of the reference drug captopril. The anti-hypertensive effect was maintained for 6 h, and the authors suggested this effect was due to the induction of NO production, which is a vasodilator [40].

#### 2.1.3. Diphlorethohydroxycarmalol

Diphlorethohydroxycarmalol **3**, which was only isolated from *Ishige okamurae* Yendo, has a protective effect against radiation exposure. Ahn et al. [46] reported that treatment with **3** (100 mg/kg b.w.) in mice before γ-ray irradiation significantly protected the intestinal crypt cells in the jejunum and maintained villi height, compared with those of the control-treated irradiated group. Mice pretreated with **3** also exhibited dose-dependent increases in the bone marrow cell viability up to a maximum of 40% at 100 mg/kg (b.w.) [46].

Diphlorethohydroxycarmalol **3** decreased the oxidative stress caused to the skin tissue of HR-1 hairless mice by fine particulate matter with a diameter ≤2.5 μm (PM_2.5_), a major pollutant present in the atmosphere [44] (Table 1). Exposure to PM_2.5_ caused lipid peroxidation and protein carbonylation, and increased epidermal height, which were inhibited by **3**. Moreover, PM_2.5_ induced apoptosis and mitogen-activated protein kinase (MAPK) protein expression; however, these changes were attenuated by **3** [44].

Fernando et al. [47] reported for the first time the use of a zebrafish embryo model for evaluating the inflammatory effects of fine dust (FD) particles, which are a major aggressive agent in air pollution. The authors determined that a concentration of 48.8 μM of **3** significantly decreased NO and ROS production and prevented fine dust inflammation-induced cell death [47]. The effect of **3** against high glucose-induced angiogenesis in zebrafish embryos was studied, and it was found that the treatment of embryos with a concentration of 2 μM of **3** suppressed high glucose-induced dilation in the retinal vessel diameter and vessel formation (Table 1). Moreover, **3** exhibits the ability to inhibit high glucose-induced vascular endothelial growth factor receptor 2 (VEGFR-2) expression and its downstream signaling cascade [48]. Hence, **3** seems to be a potential agent for the development of drugs against angiogenesis induced by diabetes.

#### 2.1.4. Eckol

Eckol **4** presented anti-inflammatory activity in various in vivo studies. Kim et al. [31] found that a concentration of 20 μM of **4** significantly suppressed acetic acid-induced hyperpermeability and carboxy-methylcellulose-induced leucocyte migration in mice at a much higher level than **1** (Table 1). A dosage of 75 nmol of **4** per mouse decreased mouse ear edema induced by different sensitizers, such as arachidonic acid (AA), 12-*O*-tetradecanoylphorbol-13-acetate (TPA), and oxazolone (OXA), by 12.7%, 40.0%, and 19.3%, respectively (Table 1) [51]. This shows that **4** can modulate various targets of the inflammatory cascade.

On the other hand, **4** at a very low dosage (0.5 mg/kg b.w.) has an hepatoprotective effect on mice by modulating anti-apoptotic and antioxidant mechanisms and suppressing the expression of pro-inflammatory cytokines, like tumor necrosis factor (TNF), interleukin (IL)-1, and IL-6, and by upregulating the expression of IL-10, an anti-inflammatory interleukin [52].

Kim et al. [53] reported that **4** presented anticoagulant activity in a mouse model. A dosage of 50 mg/kg (b.w.) increased the in vivo tail bleeding time from 51.5 to 121 s, which is an increase of more than 100%. However, this result was lower than that obtained with heparin, the commercial anticoagulant (165 s).

Eckol **4** (20 mg/kg b.w.) also significantly reduced the level of triglycerides (TG), total cholesterol (TC), atherogenic index (AI), and low-density lipoprotein cholesterol (LDL) and increased level of the high-density lipoprotein cholesterol (HDL), in SD rats, by similar values to those presented by lovastatin (Table 1), a therapeutic agent used in the treatment of hypercholesterolemia [56].

Park et al. [54] found that the administration of 10 mg/kg (b.w.) of **4** to γ-ray irradiated C57BL/6 mice led to an improvement in hematopoietic recovery and in the repair of damaged DNA in immune cells and an enhancement of their proliferation, which was severely suppressed by ionizing radiation (Table 1). It was also found that the same dose decreased lymphocyte apoptosis by 33.33% and intestinal cell apoptosis by 16.63%, which was correlated with a decrease in the amount of pro-apoptotic p53 and Bax proteins and an increase in the level of Bcl-2, an anti-apoptotic protein, indicating that its over-expression, which leads to resistance to DNA damage, is involved in protection of gastrointestinal cells after irradiation [55]. Furthermore, Moon et al. [33] found that **4** at a higher dose (20 mg/kg b.w.) enhanced jejunal crypt survival and protected against apoptosis induced by radiation in ICR mice jejunal crypts, albeit to a lesser extent than the values obtained for **1** (Table 1). These findings indicate that **4** should be a candidate for adjuvant therapy to alleviate radiation-induced injuries to cancer patients; however, as far as we were able to assess, there were no further advancements in this regard.

Pre-treatment with **4** (50 µM) reduced ROS and NO formation by about 43% and 33%, respectively, in zebrafish embryos following UV-B irradiation. It also reduced UV-B-induced cell death by 78% and hyperpigmentation by about 50%, when compared to the untreated control group, showing the photoprotection effectiveness of **4**. The compound presented low toxicity at the tested concentration [57].

#### 2.1.5. Dieckol

Dieckol **5** was able to impair the oxidative stress effects induced by ethanol in zebrafish embryos [61]. A concentration of 20 µM decreased ROS formation by 80% and lipid peroxidation by 5%. The attenuation of oxidative stress led to a 15% decrease in ethanol-induced liver cell death, showing that dieckol possesses a hepatoprotective effect [61]. Dieckol at the same dose also decreased the oxidative effects caused by high glucose, by significantly reducing heart rate, ROS, lipid peroxidation, and cell death in zebrafish (Table 1) [60]. Furthermore, high glucose levels induced the over-expression of inducible nitric oxide synthase (iNOS) and cyclooxygenase-2 (COX-2), whereas **5** treatment reduced it [60].

Additionally, the antioxidant effects of **5** also play an important role in the attenuation of type II diabetes. C57BL/KsJ-db/db diabetic mice, when injected with 20 mg/kg (b.w.) of **5**, showed a significant reduction of blood glucose level, serum insulin level, and body weight, when compared to the untreated group [58]. Nonetheless, **5** also promoted the increase of the activity of antioxidant enzymes, including superoxide dismutase (SOD), catalase (CAT), and glutathione peroxidase (GSH-px) in liver tissues, and it increased levels of the phosphorylation of AMPK and Akt in muscle tissues (Table 1), suggesting that **5** can be developed as a therapeutic agent for type II diabetes [58].

Like phlorotannin **4**, **5** also suppressed acetic acid-induced hyperpermeability and carboxy-methylcellulose-induced leucocyte migration in mice [31], albeit to a higher level than **4**, leading to the conclusion that the number of OH groups in the **5** structure increases its anti-inflammatory activity. The authors proved the influence of the OH groups of **5** on its activity by protecting those groups with a methyl substituent, and the activity obtained for methyl-dieckol was reduced by about 35% [31].

The comparison between **5** and **4** was also verified for anticoagulant activity. Kim et al. [53] found that **5** increased the in vivo tail bleeding time by 173.8%, from 51.5 to 141 s, whereas **4** only increased this time to 121 s, and heparin increased tail bleeding time to 165 s.

Dieckol also presented a better potential for treating dyslipidemia than **4** since it reduced all the parameters measured by Yoon et al. [56] at a higher level than that obtained with **4** and even lovastatin (Table 1). As an example of the efficiency of **5** in the treatment of dyslipidemia, a dose of 20 mg/kg (b.w.) of **5** decreased total cholesterol by 43.4% when compared with the untreated group, whereas lovastatin (25 mg/kg (b.w.)) only decreased this parameter by 15.3% [56].

Dieckol **5** also presents anti-allergy effects since oral administration of 5 and 20 mg/kg (b.w.), before IgE sensitization, markedly abrogated mast cell degranulation and edematous changes in vivo [59]. However, the authors also suggested that the inhibition of the passive cutaneous anaphylaxis could be mainly attributed to the anti-inflammatory effects of **5**.

#### 2.1.6. Other Phlorotannins

In the literature revision performed for the present work, phlorotannins other than those already known were found with in vitro activities reported, while they only had one or two studies addressing their in vivo activities, unlike the compounds discussed above. However, some of these activities are interesting; thus, the studies addressing the less studied phlorotannins are discussed to demonstrate the interest of future studies on these phlorotannins.

Phlorofucofuroeckol B **7** suppressed 42.2%, 38.4%, and 41.0% of ear swelling in mice induced by AA, TPA, and OXA, respectively (Table 1), whereas the suppression of ear edema induced by those three sensitizers showed was significantly lower for isomer **6** (23.4%–31.7%) [51]. This indicates that the change of the 3″,5″-dihydroxybenzyl group from C-8 in **6** to C-11 in **7** increases the compound’s anti-inflammatory capacity. The results presented by **7** were also better than those obtained for **4** (Table 1). The interesting activities shown in vivo by this phlorotannin **7** justify the realization of further studies, including more deep SAR studies to establish its action mechanism.

Administration of 6,6′-bieckol **8** (Figure 2) to mouse (75 nmol per mouse) caused the reduction of ear swelling after sensitization with AA and TPA by 41.9% and 34.2%, respectively, which is an anti-inflammatory effect similar to phlorofucofuroeckol B **7**, although **8** had a much smaller anti-inflammatory effect on the OXA-induced mouse model (17.8%) [51]. On the other hand, the administration of 6,8′-bieckol **9** was able to inhibit 77.8% of mouse ear swelling when the sensitizer was OXA, which was the highest value obtained by Sugiura et al. [51], while the administration of **10** yielded an inhibition of 32.3%. These results show clearly that the position of the linkage has a great influence on the anti-inflammatory activity of phlorotannins. Compounds **4** and **6**–**10** exhibited anti-inflammatory effects identical to or higher than epigallocatechin gallate (EGCG), the compound used as a positive control.

Ko et al. [66] found that a dose of 20 mg/kg (b.w.) of **8** led to a reduction of 28.6 mmHg in the SBP in SHR, whereas the same dosage of the reference drug captopril decreased SBP by 31.3 mmHg. This phlorotannin **8** is less active than octaphlorethol **2** [40] since the dose of **8** used was two times higher than the dose of **2** (Table 1). Thus, the latter seems to be more promising for anti-hypertensive applications.

The phlorotannin eckstolonol **11** significantly decreased sleep latency in a concentration-dependent manner and increased the amount of non-rapid eye movements (NREMS) in C57BL/6N mice by 1.4-fold at 50 mg/kg (b.w.) [67]. At this dose, **11** administered in conjunction with pentobarbital was also capable of increasing sleep duration when compared to the control (only pentobarbital), showing that this phlorotannin can also potentiate the effects of other hypnotic drugs. It was found that **11** acts as a partial agonist to the GABAA–BZD receptors [67], similar to the action mode of benzodiazepines, showing its potential as a hypnotic drug.

In addition to the good results presented by phlorotannins in in vivo studies, which showed their high pharmaceutical potential, there were some studies [31,44] where there was no information about the actual amount of compound administered, which hindered their comparison with other studies, as well as the reproducibility of the results. Also, the majority of the referenced studies, particularly those using a murine model, had a small group of individuals per study group (4–6), which may not be very representative of the real effect of the compounds. Future studies should increase the number of test subjects to increase the statistical power of the findings.

### 2.2. Peptides

#### 2.2.1. Griffithsin

**Figure 3:**
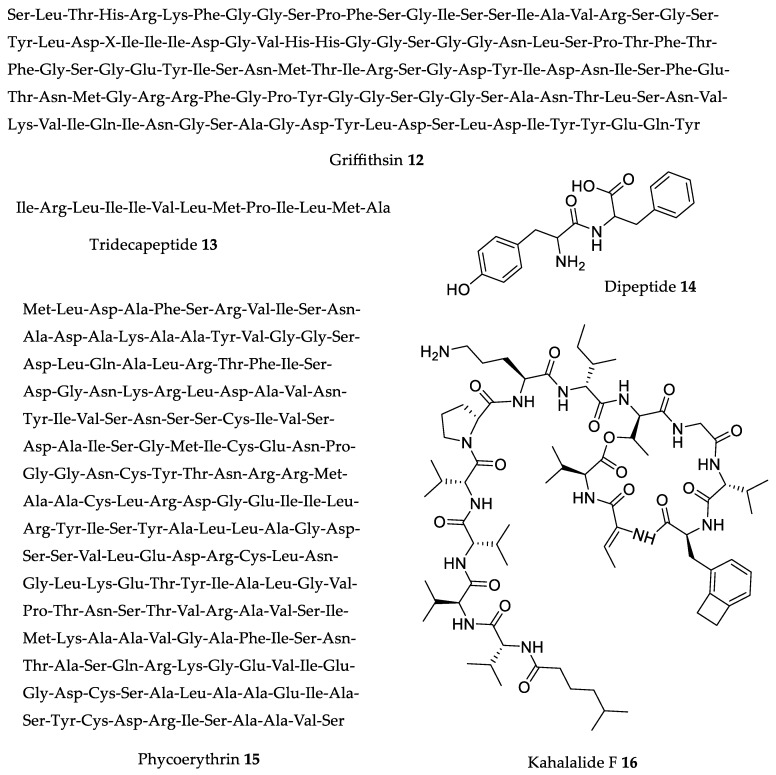
Amino-acid sequence of seaweed peptides with relevant in vivo activities.

One of the most biologically interesting families of peptides extracted from macroalgae is the lectins. They are a structurally diverse group of highly specific and reversibly carbohydrate-binding proteins [70]. The three groups of macroalgae (Rhodophyta, Phaeophyta, and Chlorophyta) can produce lectins [71], and these lectins present great potential for the development of new drugs [72,73,74,75,76]. In fact, because of the highly specific way lectins bind to sugars outside cell surfaces inhibiting cell proliferation [77,78], lectins primarily show antiviral, antibacterial, and antifungal activities [73,79,80,81]. The most interesting lectin and also the one with the most in vivo studies is griffithsin **12** (Figure 3) (Table 2). 

Griffithsin was first isolated from aqueous extracts of *Griffithsia* sp., and it exhibits antiviral activity [82]. This 121-amino-acid peptide **12** showed no significant homology (>30%) with other known proteins and exhibited potent in vitro antiviral activity (EC_50_ values ranging from 0.043 to 0.63 nM) [82], which enticed researchers to perform several subsequent in vivo studies.

O’Keefe et al. [83] reported the antiviral effect of griffithsin **12** (Figure 3, Table 2) on mouse models infected with an adapted SARS-CoV virus. After injection with a viral dose known to cause at least 75% mouse mortality, mice treated with griffithsin **12** (5 mg/kg b.w. dose intranasally delivered 4 h before infection) showed 100% survival rates, no weight loss, and decreased pulmonary pathology during infection. The compound reduced mice pulmonary viral load and inhibited the deleterious inflammatory response to the virus. In 2013, Ishag et al. [84] once again proved griffithsin’s life-saving in vivo efficacy with mice models infected with lethal doses of Japanese encephalitis virus (JEV). Similar to the results obtained by O’Keefe et al. [83], treated mice showed 100% survival rates, as well as reduced viral antigen load in brain tissue. The griffithsin **12** treatment of mice consisted of 5 mg/kg b.w. intraperitoneal injection of the encephalitis virus. The fact that the same 5 mg/kg b.w. dose was so effective against both the SARS-CoV and JEV virus highlights griffithsin’s potential as an antiviral agent. Subsequently, Meuleman et al. [85] also used a 5 mg/kg b.w. griffithsin treatment (subcutaneously injected in chimeric uPA-SCID mice) to mitigate hepatitis C liver infection. After one week of virus injection, the results showed significantly lower viral loads (below the limit of detection, <750 IU/mL) in four of the six treated mice, as opposed to easily detectable loads in the control mice. In the following two weeks of the study, while the treated mice started slowly exhibiting signs of infection, the control mice experienced full-blown viremia. Surprisingly, one of the treated mice managed to stay completely below the detection limit throughout the entire study period. These results once again point to the extent and versatility of griffithsin’s antiviral activity against taxonomically distinct viruses. Although very interesting, these results feel particularly limited in scope due to the small sample size (*n* = 6 treated and *n* = 5 control mice), a fact that was acknowledged by the authors. Nevertheless, griffithsin’s broad-spectrum antiviral action was still very “alluring” to researchers and begged further study. Nixon et al. [86] used murine models to see if a 0.1% griffithsin gel would protect mice from intravaginally applied genital herpes. Results showed that the gel significantly prevented herpes simplex virus 2 infection and proliferation after mucosal surface challenge and subsequent viral introduction in seminal plasma. These results complemented those obtained by O’Keefe et al. [87], which used the rabbit vaginal irritation model to prove griffithsin’s safety as a topical microbicide component. Results showed that griffithsin caused no mucosal damage or inflammatory responses. Another study, by Levendosky et al. [88], used a very similar intravaginal challenge methodology to assess topically applied antiviral activity of a griffithsin–carragenan (**12**-CG) combination against herpes simplex (HSV-2) and human papillomavirus (HPV16). A 20-µL dose of the combination (0.1% **12** and 3% CG) was shown to scientifically reduce HSV-2 vaginal infection (when applied before challenge) and HPV16 (when dosed during and after challenge). The discrepancy between HSV-2 and HPV16 efficacy timeframes is believed to be due to a several-hour “lag” period in HPV’s replication cycle. Notwithstanding, these results are in line with previous works and prove griffithsin’s action as a broad-spectrum antiviral. To conclude, we present a more recent study by Girard et al. in 2018 [89], who produced and rectally applied griffithsin gels in rhesus monkeys. The study confirmed the safety of griffithsin as an anti-HIV agent, with minimum disturbance of the monkey’s rectal proteome and microbiota.

In summary, griffithsin **12** shows tremendous promise as a topical antiviral agent, with great potential concerning the prevention of sexually transmitted infections. The compound’s repeatedly proven efficacy, along with the safety studies of O’Keefe et al. [87] and Girard et al. [89], appears to be leading up to a pre-clinical stage of testing, which should happen soon and eventually pave the way for future clinical trials.

#### 2.2.2. ACE and Renin Inhibitory Peptides IRLIIVLMPILMA Tridecapeptide and Phe–Tyr Dipeptide

The search for angiotensin-converting enzyme (ACE) inhibitors is of great biological value due to their inherent hypotensive effects and subsequent applications. Macroalgae were proven to be an especially rich source of compounds with ACE inhibition activity [18,99,100,101,102,103,104]. Regarding seaweed ACE or renin inhibitors, this review chooses to focus on the IRLIIVLMPILMA tridecapeptide **13** and the Phe–Tyr dipeptide **14** (Figure 3) shown in Table 2, mainly due to their potent hypotensive activity compared to a current pharmaceutical option (captopril), as well as being of more recent relevance and interest. 

Fitzgerald et al. [90] studied the hypotensive effect of the renin inhibitor tridecapeptide IRLIIVLMPILMA **13** (Figure 3), previously extracted and purified from *Palmaria palmata* (Linnaeus) F. Weber and D. Mohr hydrolysate [105], using the SHR model. The research group reported that a dose of 3 mg/kg b.w. of tridecapeptide **13** resulted in a decrease in SBP by 33 mmHg after 2 h. This is especially interesting when compared to the positive control (the clinical hypotensive drug captopril), which showed an SBP decrease by 29 mmHg with the same dose. Also noteworthy is that a 34-mmHg SBP decrease was achieved with *Palmaria palmata* (Linnaeus) F. Weber and D. Mohr protein hydrolysate but with a dose of 50 mg/kg b.w.

The SHR model was also used in a somewhat similar study by Sato et al. [91] to test ACE inhibitory peptides purified from *Undaria pinnatifida* (Harvey) Suringar hydrolysate. Seven dipeptides were identified and tested in vivo, of which the Phe–Tyr dipeptide **14** stood out, revealing a statistically significant 16-mmHg SBP decrease after 3 h with a 1 mg/kg b.w. dose and a 26-mmHg SBP decrease after 9 h with only a 0.1 mg/kg b.w. dose. These results were compared to captopril, which showed 17-mmHg and 14-mmHg SBP decreases after 3 and 9 h, respectively, with a 1 mg/kg b.w. dose. In more recent work, Kecel-Gündüz et al. [106] studied poly(lactic-*co*-glycolic acid) nanoparticles as a delivery system for the Phe–Tyr dipeptide **14**, which highlights the continued interest and relevance of this seaweed peptide with great antihypertensive potential.

As previously mentioned, of all the analyzed literature, the IRLIIVLMPILMA tridecapeptide **13** and Phe–Tyr dipeptide **14** (Figure 3, Table 2) are the most promising in vivo hypotensive seaweed compounds identified so far, with a similar effect to clinical drugs.

#### 2.2.3. Phycoerythrin

Phycoerythrin **15** (Figure 3), a red protein pigment complex abundant in Rhodophyta (although many studies use cyanobacteria as a more readily available natural source for this compound), is another polypeptide very interesting, not as a hypotensive agent but rather as an antitumor and anti-aging agent.

After extensive in vitro studies which demonstrated the cytotoxic activity of phycoerythrin **15** (Figure 3) [94,107], Pan et al. [92] demonstrated its activity in vivo using the S180 tumor-bearing mice model (Table 2). Results showed that phycoerythrin injection, at a dose of 300 mg/kg b.w., reduced S180 tumor growth by up to 41.3% in treated mice. These mice also revealed a significant serum increase in the TNF-α level, NK cell kill activity, and lymphocyte proliferation. The antitumor activity obtained is believed to be related to phycoerythrin’s antioxidant activity, as shown by the significant increase in superoxide dismutase activity in the serum of treated mice, as well as the significant decrease in mouse liver malondialdehyde level.

Shortly after, Sonani et al. [95] used the *Caenorhabitis elegans* model to test the in vivo antioxidant and anti-aging effects of phycoerythrin. Doses of 100 μg/mL of the compound were found to significantly increase *Caenorhabitis elegans* lifespan both in normal and in oxidative stress conditions. This is indicative of phycoerythrin having a strong anti-aging effect, possibly related to its antioxidant properties.

In more recent work, Chaubey et al. [96] tested the effect of phycoerythrin in a mutant *Caenorhabitis elegans* Alzheimer’s disease model. Results showed that a dose of 100 μg/mL of phycoerythrin led to a significant reduction in senile plaque formation when compared to untreated nematodes. This indicates that phycoerythrin might have great potential as a therapeutic agent in neurodegenerative diseases, but more tests are required to confirm this.

#### 2.2.4. Kahalalide F

Kahalalide F **16** (Figure 3) is a cyclic depsipeptide that belongs to the kahalalide protein family. It was first described by Hamann and Scheuer [97], isolated from *Bryopsis* sp. green alga, as well as from the *Elysia rufescens* mollusk, which feeds on *Bryopsis* and bio-accumulates **16** (which is why most studies used the mollusk as a source of this compound). In vitro studies [108] revealed the great cytotoxic potential of **16** against several tumor cell lines, particularly prostate and breast cancer lines, with IC_50_ values ranging from 0.07 to 0.28 μM [109]. In vivo studies carried out by Faircloth and Cuevas [98] showed the tumor response to injected **16** (Figure 3, Table 2) in human breast, prostate, colon, and lung tumor cells xenografted into athymic mice. Treatment with a 0.245 mg/kg (b.w.) dose led to ~50% smaller chemotherapy-resistant DU-145 prostate tumor, while the PC-3 human prostate tumor was reduced by nearly 35% with a 0.123 mg/kg (b.w.) dose. These highly promising results led kahalalide F **16** to the clinical trial phase, which is discussed later.

### 2.3. Halogenated Secondary Metabolites

Halogenated compounds are also an interesting set of bioactive macroalgae secondary metabolites [17,110,111,112,113]. Among these, halogenated terpenes and bromophenols are those whose in vivo studies revealed the greatest potential for new drug development, as discussed below.

**Figure 4:**
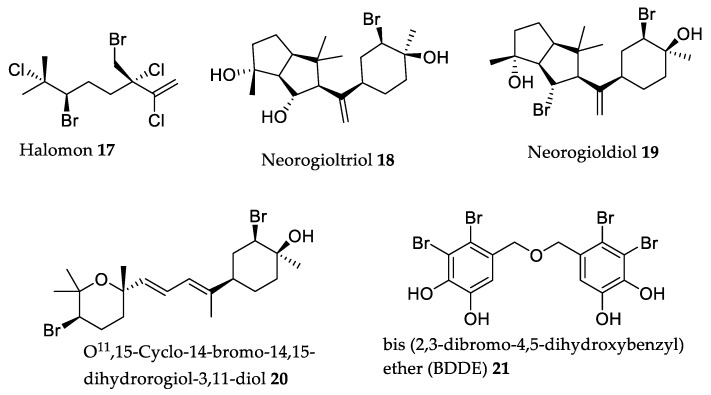
Chemical structure of some halogenated compounds.

Pentahalogenated monoterpene 6*R*-bromo-3*S*-(bromomethyl)-7-methyl-2,3,7-trichloro-1-octene, known by trivial name halomon **17** (Figure 4, Table 3), showed the most promise in in vitro cytotoxic studies (sub-micromolar IC_50_ values) [17], going so far as to be selected by the National Cancer Institute within the NCI60 human tumor cell line anticancer drug screen program, for preclinical studies for drug development. Although this testing never went beyond a preliminary phase, the first results were very promising, showing 40% of “apparent cures” of a very aggressive U251 brain tumor in mouse ip/ip xenograft models [114].

The latest published results regarding this preclinical trial process were related to halomon **17** tested in CD_2_F_1_ mice regarding bioavailability, pharmacokinetics, and tissue distribution [123]. The results showed that halomon bioavailability is higher after intraperitoneal injection and subcutaneous injection (45% and 47%, respectively), while its urinary excretion is minimal. Halomon **17** is distributed in all tissues but with a higher concentration in adipose tissue. The concentration of halomon measured in the brain is comparable to that detected in plasma and most other tissues. Even though preclinical testing never progressed beyond preliminary stages, this never deterred the scientific community’s interest in **17** over time, and a more recent study about the action mechanism of **17** proposed that it acts as a DNA methyl transferase-1 inhibitor [124]. However, more deep mechanism studies should be performed.

In addition to these studies, a real obstacle to overcome with halomon **17** is always to obtain enough quantity of the compound. Fuller et al. [114] described this struggle by stating that “slight geographic and/or temporal change” would dramatically affect the terpene content of *Portieria hornemanii*, and that alternative approaches should be considered. Naturally, this problem led to chemists trying to synthesize halomon, with the first success occurring in 1998 by Schlama et al. [125], who reported a 13% overall yield. A result was obtained by Sotokawa et al. [126], reducing the previous 13-step process into three steps, reporting a 25% overall yield but with poor selectivity. Only in 2015 was the first efficient and high-selectivity method described by Bucher et al. [127], a process which was since further optimized by Landry and Burns [128]. Having finally overcome this obstacle after over 25 years, halomon **17** in vivo studies should be restarted as to finally confirm its potential.

Another highly interesting compound is neorogioltriol **18** (Figure 4, Table 3), a tricyclic brominated diterpenoid first isolated from *Laurencia glandulifera* by Chatter et al. [115]. This research group showed that neorogioltriol had analgesic properties. In the writhing test, neorogiotriol produced a dose-dependent response, and a dose of 1 mg/kg (b.w.) was enough to reduce the mouse acetic acid-induced writhing response by 88.9% (Table 3). With the rat model, the formalin test was used to determine if the compound affected neurogenic and/or inflammatory pain. Results showed that neorogiotriol **18** reduced licking time by 48%, but only in the second phase of the formalin test, indicating that the compound has a peripheral analgesic effect, acting on inflammatory pain in a way typical of cyclooxygenase inhibitors. Chatter et al. [115] supplemented their previous work with neorogioltriol **18** by testing its in vivo anti-inflammatory effect on induced rat paw swelling. Results showed that an injected dose of 1 mg/kg (b.w.) of the compound reduced paw swelling by 28% after the first hour and 58% after three hours. To achieve the same anti-inflammatory result with a reference compound, acetylsalicylic acid would require a dose of 300 mg/kg (b.w.) [115].

A more recent paper published by Daskalaki et al. [117] studied two diterpenes, neorogioldiol **19** and O^11^,15-cyclo-14-bromo-14,15-dihydrorogiol-3,11-diol **20** (Figure 4 Table 3). These compounds were used to treat C57BL/6 mice with DSS-induced inflammatory bowel disease (colitis). A 0.25 mg/mouse dose of each compound was intraperitoneally injected every 48 h, in two different groups. The results showed that treated mice demonstrated reduced inflammatory colonic tissue damage, as well as a very significant decreased of pro-inflammatory cytokine messenger RNA (mRNA) (more than 40-fold decrease in the case of interleukin-6). Neorogioldiol **19** and O^11^,15-cyclo-14-bromo-14,15-dihydrorogiol-3,11-diol **20** showed similar activity levels and revealed their great potential for bowel disease inflammatory treatment. More studies should be pursued, particularly to assess the neorogiotriol **18** activity in the previously mentioned colitis model once it is structurally related to compounds **19** and **20**.

Bromophenols are another class of very interesting macroalgae metabolites. Although most studies of this family of compounds only showed in vitro effects so far, a few of them reached the level of being evaluated in an in vivo model. One of the most biologically relevant of such compounds is BDDE **21** (Figure 4, Table 3).

First isolated by Kurihara et al. in 1999 from Rhodophyta *Odonthalia corymbifera*, these researchers showed BDDE **21** as an α-glucosidase inhibitor [119]. After this, some very promising in vitro studies confirmed **21**’s α-glucosidase interaction [129] and showed **21**’s anticancer [120,130] and antifungal activities [131]. A recently published study [122] showed that **21** had in vivo antidiabetic activity. The research group showed that a dose of 40 mg/kg (b.w.), orally administered, was more effective at lowering blood glucose levels in db/db mice than metformin (a clinical antidiabetic drug). The study also showed that **21** significantly reduced glycated hemoglobin, triglyceride levels, and body weight without influencing the mice’s food or water intake. This shows that **21** might constitute a powerful antidiabetic drug in the future, but more testing is required to ascertain this possibility. Another interesting in vivo study, using a different animal model, was also published in 2015 by Qi et al. [121], revealing a different effect. In this work, BDDE **21** exhibited potent angiogenesis inhibition activity in zebrafish embryo models [121]. In this work, researchers monitored the embryonic development of the zebrafish sub-intestinal vessel (SIV) when incubated in the presence of **21**. Results showed a statistically significant and dose-dependent response, with 6.25, 12.5, and 25 mM reducing SIV growth by 17.7%, 40.4%, and 49.5%, respectively. This unequivocally proves **21**’s effect as an anti-angiogenesis agent and points to its great potential for cancer therapeutic applications; however, more in vivo antitumor studies are necessary. In summary, there is a considerable diversity of algae halogenated secondary metabolites with very interesting and promising bioactivities, which might lead to future drug developments; however, more testing is required.

### 2.4. Fucoxanthin

**Figure 5:**
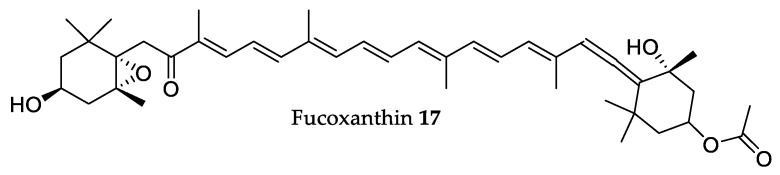
Chemical structure of fucoxanthin.

Concerning algal lipids, fucoxanthin **22** (Figure 5), a xanthophyll-like carotenoid, is one of the most studied metabolites because of its beneficial health effects [18,103,132]. Indeed, there are many published reviews and research articles demonstrating and extolling, among others, the nutraceutical, antioxidant, anticancer, anti-obesity, antidiabetic, antimicrobial, and cardiovascular protective effects of fucoxanthin **22** [103,132,133,134,135,136,137,138,139].

It is intended here to review the most relevant in vivo studies with pure fucoxanthin, highlighting the impact that each one had on the process of development of fucoxanthin as a drug with many potential therapeutic uses. 

Fucoxanthin **22** (Figure 5) seems to have a neuroprotective effect, as evidenced by Hu et al. [140] using the middle cerebral artery occlusion rat model (MCAO) [141]. To assess a neuroprotective effect, the rats were intragastrical administered different doses (30, 60, and 90 mg/kg b.w.) of pure fucoxanthin 1 h before cerebral ischemia was induced. Results showed significant and dose-dependent reductions of neurological deficit scores and percentages of infarcted area in the brain, as well as an attenuation of brain edema. One criticism that could be made of the researchers’ work pertains to how they presented the objective results of their essays; the results were presented only in graph form with no supporting table listing the values. This makes it hard to properly and objectively assess the degree to which the neurological parameters tested showed an improvement or not. Nonetheless, the published work did serve to firmly support fucoxanthin as a potential neuroprotective supplement of interest.

Another highly interesting potential pharmaceutical application for fucoxanthin was illustrated in the recently published work by Wang et al. [142], which reports fucoxanthin antitumor activity in a novel lymphangiogenesis inhibition perspective. In this work, the MDA-MB-231 breast cancer xenograft model was used on Balb/c nude mice treated with 6.58 and 32.9 μg doses of **22**. Fucoxanthin was injected daily on the tumor periphery, and tumors were excised after 26 days. Results revealed significant decreases in micro-lymphatic vascular density, from an average of 14.0 ± 2.94 lymphatic vessels to 6.0 ± 0.81 (with 6.58 μg fucoxanthin treatment) and 3.66 ± 1.25 (with 32.9 μg treatment) per tumor. Tumor weight and volume also decreased by more than half in a dose-dependent manner, although, once again, it is difficult to assess this reduction precisely due to the lack of a values table accompanying the results graph. However, these results adequately highlight **22**’s potential in cancer treatment. In another 2019 study by Terasaki et al. [143], this anti-tumor activity was again tested, this time with a colorectal cancer mouse model. In this work, AOM/DSS mice were injected with a 30 mg/kg (b.w.) daily dose of fucoxanthin oil for seven weeks, with subsequent bowel excision and analysis post sacrifice. Results showed that **22** significantly reduced the number of colonic polyps by close to half compared to non-treated mice, with polyp size also significantly reduced to about one-third of the control mice. Objective histological examination showed a reduction in the prevalence of tumors, ulcers, and crypt dysplasia. The authors suggested that this may be linked to **22** promoting anoikis-like cell death, and they supported this hypothesis by showing increased expression (2–5-fold) of key molecular hallmarks for anoikis in treated mice colon cells. These results reinforce **22** as a good candidate for possible anti-cancer drugs. In addition to this bioactivity, a 2019 paper by Jiang et al. [144] highlighted **22**’s potential as an antidepressant. In this work, a lipopolysaccharide-induced depressive-like behavior mouse model was used, to evaluate if **22** treatments would reduce depressive or anxiety associated behaviors. Results showed that treated mice had significantly higher body weight and food intake than control mice, as well as significantly reduced depressive-like behavior and anxiety-like behavior. These behaviors were assessed by presenting the mice with stressful conditions/obstacles and then evaluating their activity. It is important to note that the lower doses of **22** used in this work showed a very marginal depressive behavioral reduction, but the highest dose tested (200 mg/kg b.w.) managed to reduce depressive and anxiety-like behaviors to almost baseline values of non-depressed mice. In other words, a 200 mg/kg (b.w.) dose of **22** significantly reduced depressive behavioral traits to the point where the induced depression was practically “cured”. While this dosage is considerably higher than that used in previously mentioned studies, we chose to highlight this neuroprotective bioactivity here due to its novelty and relative relevance.

To finalize, another 2019 study by Su et al. [145] revealed that **22** has great potential as an anti-inflammatory in a mouse sepsis model. In this work, lipopolysaccharides were once again used (albeit at a much higher dose than in the previous study) to induce sepsis, eventually leading to death in the mouse models. The results showed that, while a 10 mg/kg (b.w.) dose of LPS caused a 20% survival rate in the mice, the same dose in mice treated with 1 mg/kg (b.w.) of **22** had a 40% survival rate. A single very small dose of **22** injected 30 min prior to challenge effectively doubled the survival rate of the sepsis mouse model. In addition, treated mice also showed significantly reduced levels of pro-inflammatory cytokines TNF-α (~30% reduction) and IL-6 (~90% reduction) when compared to non-treated mice, as well as significantly inhibiting the NF-κB inflammatory pathway (as shown by the ~50% reduction in p-IκBα, and p-NF-κB). This shows that **22** exhibits a potent anti-inflammatory effect and can effectively have a strong protective effect in an acute inflammatory disease model. In summary, **22** exhibits a multitude of very interesting and diverse potent bioactivities, with studies very recently published. The scientific community appears to have a great interest in this compound, and we hope to see more high-quality in vivo publications in the near future.

### 2.5. Fucosterol

**Figure 6:**
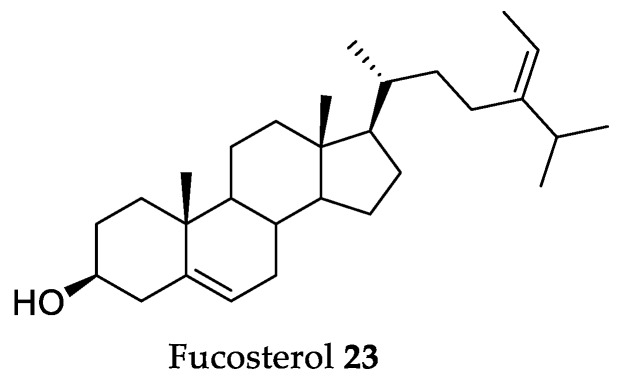
Chemical structure of fucosterol.

Fucosterol **23** (Figure 6) is a phytosterol, mostly isolated from brown algae, and it is relatively abundant in this particular algal class. It was widely studied regarding its in vitro health effects [146]; however, in vivo evaluations of fucosterol’s health effects are very scarce. In this regard, the present work reviews the existing in vivo studies, and the main observations and conclusions are discussed in the paragraphs below.

One of the first evaluations of the in vivo effects of fucosterol **23** was regarding its anti-diabetic effects, and it was found that, when administered orally at 30 mg/kg in streptozotocin-induced diabetic rats, fucosterol caused a significant decrease of 14.8% in serum glucose concentrations, and exhibited an inhibition of sorbitol accumulations in the lenses of 22.4% when compared to the untreated group [147].

This phytosterol presents antitumor activity in vivo, with a dosage of 40 mg/kg (b.w.), reducing about 75% of tumor weight and 50% of tumor volume after six weeks in lung cancer xenografted C57 BL/6 mice model [148]. In addition, fucosterol **23** (40 mg/kg b.w.) reduced Ki-67 expression, an indicator of cell proliferation, by 60%, and increased cleaved caspase-3 levels by more than 100%, which indicates that **23** acts in the tumor cells by simultaneously decreasing their proliferation and enhancing their apoptosis [148].

Fucosterol **23** also exhibits a protective effect on LPS-induced acute lung injury (ALI), by modulating the expression of pro-inflammatory factors [149]. A dosage of 30 mg/kg (b.w.) of **23** attenuated lung histopathologic changes and the wet-to-dry ratio of lungs in LPS-induced ALI in mice. Furthermore, fucosterol significantly inhibited TNF-a, IL-1ß, and IL-6 levels in both the broncho-alveolar lavage fluid (BALF) and the LPS-stimulated alveolar macrophages, reducing their expression by about 50%, when compared to the untreated group [149]. The fact that **23** is able to inhibit the production of pro-inflammatory molecules suggests that it could be used for the treatment of other inflammatory diseases. This suggestion was confirmed by the findings of Mo et al. [150], where it was observed that fucosterol **23** attenuated serum liver enzyme levels, hepatic necrosis, and apoptosis induced by TNF-α, IL-6, and IL-1β. In fact, a dosage of 50 mg/kg (b.w.) of fucosterol reduced the serum levels of these three pro-inflammatory molecules by 37.5%, 31.3%, and 33.3%, respectively, after 8 h of exposure to concanavalin-A, the inducer of acute liver injury. The authors also found that **23** (50 mg/kg b.w.) also inhibited apoptosis and autophagy by upregulating Bcl-2 (12-fold increase), which decreased levels of functional Bax (50%) and Beclin-1 (46%). Furthermore, reduced P38 MAPK and NF-κB signaling were accompanied by PPARγ activation, showing that fucosterol acts by inhibiting P38 MAPK/PPARγ/NF-κB signaling [150].

Fucosterol **23** is able to reduce the effects of postmenopausal osteoporosis. A study performed with ovariectomized rats found that the bone mineral density of femoral bones was significantly higher in **23** (50 mg/kg b.w.) treated groups than in the untreated group [151]. Additionally, body weight after six weeks of treatment was 6% lower in the fucosterol **23** treated groups, when compared to the untreated group. In terms of serum biomarkers of bone formation and resorption, **23** (100 mg/kg b.w.) tripled the level of serum osteocalcin relative to the untreated group and reduced the serum level of CTx by 60%, which suggests that fucosterol **23** has the potential to activate osteoblasts, stimulate bone formation, suppress differentiation of osteoclasts, and reduce bone resorption [151].

In terms of neurological effects of fucosterol **23**, this compound was found to attenuate sAβ_1-42_-induced cognitive impairment in aging rats [152]. In fact, aged rats treated with only sAβ_1-42_ performed poorly in acquisition training and memory tests, whereas co-infusion of 10 µmol/h of **23** for the four weeks of assay restored the rats’ performance to the level of the healthy control. Fucosterol **23** action was via downregulation of GRP78 expression and upregulation of mature brain-derived neurotrophic factor (BDNF) expression in the dentate gyrus, which means it is able to suppress aging-induced endoplasmic reticulum (ER) stress [152].

Fucosterol-induced upregulation of BDNF levels is also linked to other neurological actions, like antidepressant activity. In fact, **23** (20 mg/kg b.w.) administration to Balb/e mice reduced immobility time in the forced swim test, which is a measure of depression, by 82.2 s, a value very similar to that obtained with the positive control, fluoxetine, at the same concentration (85.1 s) [153]. The same effect was observed in the tail suspension test, where both fucosterol **23** and fluoxetine (20 mg/kg b.w.) significantly shortened immobility time in the forced tail suspension test by approximately 80 s, when compared with the untreated group. Fucosterol **23** (20 mg/kg b.w.) significantly increased serotonin, norepinephrine, and the metabolite 5-hydroxyindole acetic acid in the mouse brain, with levels very close to that observed in the brain of mice not subjected to the stress of the tail suspension and forced swimming tests. This suggests that the effects of fucosterol **23** may be mediated through these neurotransmitters [153]. Also, a significant increase in hippocampal brain-derived neurotrophic factor (BDNF) levels was found in the fucosterol 20 mg/kg (b.w.) group, which suggests that the antidepressant effect may be mediated by increasing central BDNF levels [153].

The findings presented show that **23** could be an efficient therapeutic agent for a wide array of health conditions. Regardless, the number of in vivo tests existing with this algal metabolite is still very scarce; thus, we suggest that future works should invest in assessing the full in vivo potential of fucosterol **23**.

## 3. Clinical Trials

The above-mentioned information regarding the performance of seaweed compounds and derivatives in the in vivo assays shows that these types of compounds have great pharmaceutical potential with some of them already being in clinical trial phases.

Fucoxanthin **22** (Figure 4) is one of them, with two studies scheduled to begin at the end of 2019, one a phase II study that aims at fucoxanthin’s effects on metabolic syndrome (ClinicalTrials.gov identifier: NCT03613740) and the other that will test an oral dietary supplement rich with fucoxanthin for improving liver health (ClinicalTrials.gov identifier: NCT03625284).

Additionally, some trials already reached the end and presented their results. Hitoe and Shimoda [154] reported that a month of treatment with 3 mg of **22** per day had weight loss effects in mildly obese Japanese adults (BMI > 25 kg/m^2^) since it reduced abdominal fat, body weight, and overall BMI compared to the placebo group. These results are in accordance with those described by Abidov et al. [155] who performed a 16-week clinical trial in 151 women using a dietary supplement named Xanthigen composed of pomegranate seed oil and brown seaweed extract containing 2.4 mg of **22**, which increased resting energy expenditure, and induced body fat reduction and weight loss in obese women (BMI > 30 kg/m^2^).

Kahalalide F **16** (Figure 3), as already mentioned in Section 2.2.4, is a promising peptide that is being tested in clinical trials, particularly for its antitumor properties. Martín-Algarra and colleagues [156] investigated the response of patients with advanced malignant melanoma to **16**, through weekly intravenous administration of 650 μg/m^2^ until patient refusal, unacceptable toxicity, or disease progression was observed. The results indicated that, contrary to the majority of other chemotherapeutic agents, **16** did not induce severe cardiac, renal, or bone marrow toxicity, alopecia, diarrhea, or mucositis, and it was able to stabilize the disease for more than three months in five of 21 patients (23.8%) who completed the study.

A more recent study [157] evaluated the **16** weekly intravenous administration maximum tolerated dose and infusion times to recommend appropriate doses and treatment times for further phase II clinical studies in patients with advanced solid tumors. Based on the results, the authors recommended a dose of 1000 μg/m^2^ of **16** with three hours of treatment per week; however, prolonged infusion times (i.e., 24-h treatment) are also feasible.

Unfortunately, only these two compounds from all those mentioned in Section 2 reached clinical trials, which could be due to diverse complications like obtaining the necessary approvals required to start the study, obtaining volunteers, or isolating the compound of interest in sufficient quantities to allow the studies to unfold.

On the other hand, since seaweeds represent a good source of compounds with pharmaceutical potential and since seaweeds are attaining more interest in Western countries’ diets, the majority of clinical trials are currently carried out to ascertain to what extent the consumption of algae improves human health. Thus, the clinical trials discussed below focused on testing the effects of consuming one type of seaweed (or a mixture of them) or its various rich fractions/extracts.

With a quick search on ClinicalTrials.gov, it is possible to find 25 clinical trials that were seaweed-relevant. From those 25 clinical trials, two are active and ongoing, and six are scheduled to start shortly, which shows the current interest and relevance of this topic. Unfortunately, from the 17 already completed clinical trials, only eight had their results published. Additionally, it was possible to find other clinical trials that were not listed on this database, and which contributed also to an overview of this topic with growing interest.

Several clinical studies aimed at evaluating the effect of polysaccharide fractions, extracts, and even whole seaweed on the treatment and prevention of diabetes and obesity. These important aspects that are beyond the scope of this review topic, but we refer our readers to interesting publications about this subject [158,159,160,161,162,163].

A recent study conducted by Murray et al. [164] found that a single dose up to 2000 mg of a polyphenol-rich *Fucus vesiculosus* Linnaeus extract had no additional lowering effect compared to placebo on postprandial blood glucose or plasma insulin in healthy adults. The authors suggested that future studies with polyphenol-rich marine algal extracts should aim to investigate the glycemic modulating effects in at-risk populations, such as pre-diabetics, since the results may be different.

Another clinical trial from the same year [165] examined, in 60 healthy adults, the effect of brown seaweed extract InSea2^®^ consumption on their postprandial cognitive function. A dose of brown seaweed extract (500 mg), containing 20% phlorotannins, was consumed 30 min before lunch. Attention, episodic memory, and subjective state were the parameters analyzed five times over a 3-h period following lunch with 40-min intervals between measures. The results demonstrated an improvement in cognitive performance following the ingestion of the seaweed extract when compared to the placebo group since accuracy was increased in the choice reaction time and on the digit vigilance tasks. The authors [165] pointed out that, since the brown seaweed extract was a supplement equivalent of 10 g of dried seaweed, the cognitive benefits presented in this work could be obtained from dietary intake of seaweed consumption.

Regarding seaweed consumption, another study [166] investigated the acceptability of *Ascophyllum nodosum* (Linnaeus) Le Jolis-enriched bread as part of a meal by overweight healthy males, to see if it could modulate cholesterolemic and glycemic responses and reduce energy intake. Four hours after the enriched bread consumption at breakfast (using a test meal), the energy intake suffered a significant reduction (16.4%). According to the study results, it is acceptable to incorporate this seaweed into a basic food such as bread, at least at concentrations of up to 4% wholemeal loaf. Considering the interesting results of this acute feeding trial, the authors accentuated that a long-term study regarding the addition of seaweed-enriched bread to diets of participants would help to clarify its potential for the reduction of energy intake, potentially positively affecting their body mass index (BMI).

Higher oxidant status increases the oxidative damage of macromolecules, which, associated with obesity, increases the probability of chronic disease development [167], with obese individuals as a risk group. Baldrick et al. [168] investigated the bioavailability and effect of an *Ascophyllum nodosum* (Linnaeus) Le Jolis polyphenol-rich extract on DNA damage, oxidative stress, and inflammation level. Eighty participants, of which 36 were obese, consumed daily, for eight weeks, a capsule containing 100 mg of *Ascophyllum nodosum* (Linnaeus) Le Jolis polyphenol-rich extract. After the trial period, only the obese individuals presented results significantly distinct from placebo, with a 23% decrease in lymphocyte DNA damage. Thus, this work suggests that long-term consumption of *Ascophyllum nodosum* (Linnaeus) Le Jolis polyphenols rich extract could be beneficial since it could potentially decrease the risk of chronic disease development in obese individuals.

In other lines of research, Allaert et al. [169] found that, when compared with a placebo, a daily intake of a water-soluble extract of *Ulva lactuca* Linnaeus (6.45 mg per kg body weight) for three months significantly improved the depression state of subjects presenting anhedonia (a loss of sensitivity when it comes to feeling pleasure). In the placebo group, 72.5% of participants said they felt an improvement in mood versus 90.1% of the participants in the *Ulva lactuca* Linnaeus extract group (a statistically significant difference). Similarly, 70.8% of doctors judged the subjects in the placebo group to have improved versus 90.9% of the participants in the *Ulva lactuca* Linnaeus extract group. As the authors pointed out, identifying the compound in the seaweed extract responsible for the witnessed effect in this work opens up perspectives for its potential use in depression therapy.

Teas and Irhimeh [170] showed a synergistic effect between the daily consumption of brown seaweed (*Undaria pinnatifida* (Harvey) Suringar) (2.5 g) and spirulina (*Arthrospira platensis* Gomont) (3 g), since it was able to increase immune response and decrease HIV viral fusion/entry and replication in a three-month period. Furthermore, one subject continued in the trial for 13 months and reported decreased HIV viral load (from 3.3 to 2.8 log_10_) and clinically significant improvement in CD4 (>100 cells/mL). Despite the promising results, it should be noted that the sample size in this work was too small (*n* = 11) to make any generalizations about the efficacy, and further research is imperative.

Since higher levels of serum estradiol (E2) are associated with an increased risk of breast cancer development [171], Teas et al. [172] reported that a daily dose of 5 g of *Alaria esculenta* (Linnaeus) Greville for seven weeks had the ability to modulate serum hormone levels and urinary excretion of estrogen metabolites and phytoestrogens, diminishing breast cancer risk in women. Again, the conclusion of this study was limited by the small number of participants (*n* = 15), which limited the statistical power of the results.

The results of the various clinical trials mentioned above point out that the consumption of algae, particularly brown algae, can be beneficial to human health. However, in our opinion, it is also necessary to perform the identification of the chemical compounds responsible for the observed effects. There are several studies where the authors did not relate the observed effect to any constituent of the seaweed/extract evaluated, and having studies with fractions rich in a given class of compounds does not substitute for the identification of the bioactive metabolites and their health effects. Nevertheless, these studies are also important because they established that some seaweeds can be used for human consumption.

## 4. Critical Opinion

In the last few years, secondary metabolites isolated from macroalgae gained growing interest, as shown by the numerous articles reporting in vivo studies, with some compounds reaching clinical trial phases. Although many studies presented their results with quality, there were some points that deserve to be highlighted regarding the majority of the consulted papers.

Future in vivo studies, especially those with murine models, should increase the number of individuals for each test group to increase the statistical power of the findings. Also, a reference compound should always be used, to assess the real efficacy of the tested compounds. The frequent lack of clarity in result presentation in several publications was also a downside in interesting and promising studies.

Clinical trial studies with isolated compounds, unfortunately, are scarce. This could be due to diverse complications like obtaining the necessary approvals required to start the study, obtaining volunteers, or obtaining the compound in enough quantities. Additionally, most of the clinical trials aimed at ascertaining to what extent the consumption of algae, as a whole or as extracts or fractions, affects human health, particularly the effects regarding obesity and diabetes. Nonetheless, it is unfortunate that many of the mentioned studies were carried out with such small population samples, which deprives them of statistical power. Another serious flaw in the numerous studies addressing algae extracts is the fact that their chemical composition was not mentioned or is unknown, and extrapolation of the effects of extracts or algae on their secondary metabolites is in no way guaranteed and/or valid. The knowledge of the bioactive metabolites and their activity is important but does not validate the algae’s consumption.

Despite the indicated limitations, these extract clinical trials are relevant for qualitative and safety evaluations. In our opinion, these studies will also contribute to the scientific community’s interest, resulting in a deeper analysis that will uncover the most active metabolites.

Regardless of that, in most cases, these studies represent the first steps on the way to enhancing algae’s potential as a pharmaceutical source of new compounds with promising properties.

## 5. Conclusions

Phlorotannins show great pharmaceutical potential in vivo. Most of the studies indicated that the main sources of bioactive phlorotannins are algae from the *Eisenia*, *Ecklonia*, and *Ishige* genera. However, this observation can be the result of the studies’ geographical distribution. The studies reviewed herein showed that phlorotannins’ mechanisms of action are mainly related to the modulation of oxidative stress and the inflammatory cascade. Phloroglucinol **1**, eckol **4**, and dieckol **5** are compounds with a wide range of applications. The dieckol **5** anti-dyslipidemia activity must be highlighted because it is more effective than lovastatin, the clinically used drug. The hepatoprotective activity of eckol **4** should also be emphasized, since a very low dose (0.5 mg/kg b.w.) is needed.

Concerning other non-phlorotannin groups of compounds, it is clear that there is a great variety of very interesting compounds, with many of them in dire need of further testing. Out of these, the bioactive effects of the peptides griffithsin **12**, tridecapeptide IRLIIVLMPILMA **13**, and kahalalide F **16** should be highlighted, as they are arguably the most promising of all non-phlorotannins. Kahalalide F already moved beyond the in vivo stage to clinical trials, whereas tridecapeptide **13**, with a level of activity similar to the clinical drug captopril, and griffithsin **12**, which showed such stunning results over a variety of animal models, will probably move into the clinical trial stage soon. In contrast, there are promising compounds such as halomon **17** and neorogioltriol **18**, which exhibited potent and very relevant bioactivities and were not subjected to clinical trials. Hopefully, the discussion presented in this paper about their activities will interest the scientific community, and further studies will be conducted.

Regarding the fact that clinical trials with isolated compounds are scarce, only those carried out with kahalalide F **16** and fucoxanthin **22** were found, whereas we also analyzed a few clinical trials involving seaweed extracts. It can be concluded that the consumption of brown algae can be beneficial to human health, with *Ascophyllum nodosum* (Linnaeus) Le Jolis as the leading seaweed in clinical trials.

## Figures and Tables

**Table 1:** Summary of in vivo activity of phlorotannins.

Compound	Source	Model	Dose	Activity
Phloroglucinol **1**	*Eisenia bicyclis* (Kjellman) Setchell [31], *Ecklonia cava* Kjellman [32,33,34]	ICR mice	20 μM	Suppression of acetic acid-induced vessel hyperpermeability (20%) and CMC-induced leucocyte migration (36.4%) [31].
		Balb/c mice	50 and 100 mg/kg (b.w.)	Protects against γ-radiation damage increasing survival rate (70% and 90% against 40% in the control group, observed 30 days after exposure to lethal doses of ionizing radiation) [32].
		Balb/c mice	25 mg/kg (b.w.)	Reduction of breast tumor growth by 82% compared to untreated group [35].
		NOD scid gamma mice	25 mg/kg (b.w.)	33.3% less metastasis of breast cancer cells and extended survival rate (40% after 10 weeks against 0% untreated group) [36].
		C57BL/6J mice	100 mg/kg (b.w.)	13% improvement in glucose tolerance compared to untreated group. 60% inhibition of glucose synthesis in primary mouse hepatocytes [37].
		ICR mice	20 mg/kg (b.w.)	Enhanced jejunal crypt survival (26.4%) and reduction of apoptotic cells (32.5%) in jejunal crypts after γ-ray exposure [33].
		HR-1 hairless mice	100 mg/kg (b.w.)	High reduction of UV-B-induced wrinkle formation (25%), epidermal thickness (62%), and elastic fiber degeneration (75%) when compared with control group [38].
		Balb/c mice	10 mg/mouse * (topical application)	Protection against UV-B-induced DNA damage by induction of NER pathway: Increase of 50% in XPC expression and of 66% in ERCC1 expression [39].
		Zebrafish embryos	50 μM	Reduction of H_2_O_2_ induced oxidative stress damage, with survival rate of 90% against 60% in untreated group [34].
Octaphlorethol A **2**	*Ishige sinicola* (Setchell and N.L. Gardner) Chihara [40], *Ishige foliacea* Okamura [41,42,43]	SHR rats	10 mg/kg (b.w.)	Reduction of 21.9 mmHg in systolic blood pressure against 26.3 mmHg obtained with captopril [40].
		Zebrafish embryos	50 μM	Decrease glucose-induced ROS generation (10%) and lipid peroxidation (20%). Increase survival rate (50%) [41].
		Zebrafish embryos	12.6 μM *	Decrease of AAPH-induced ROS formation (30%) and lipid peroxidation (25%) when compared with the untreated group. Toxic at concentration higher than 50.4 μM [42].
		Zebrafish embryos	25 μM	Inhibition of melanin synthesis (27.8%) and tyrosinase activity (32.8%) Inhibitory activity higher than arbutin at 500 μM [43].
Diphlorethohydroxycarmalol **3**	*Ishige okamurae* Yendo [44,45]	HR-1 hairless mice	2 mM	Inhibition of PM_2.5_ exposure-induced lipid peroxidation (25%), protein carbonylation (37.5), and epidermal height (12%) [44].
		Balb/c mice	100 mg/kg (b.w.)	Protection against radiation-induced cell damage and increase by 30% in number of crypt cells compared with untreated group. Maintained villi height. Reduction of 50% of lipid peroxidation in liver. Bone marrow cell viability increased (40%) [46].
		Zebrafish embryos	48.8 μM *	Decrease of fine-dust particle-induced NO (50%) and ROS production (32%). Decrease inflammation-induced cell death (40%) [47].
		Zebrafish embryos	2 μM	Suppression of high glucose-induced dilation in the retinal vessel diameter (64.9%) and vessel formation (35.6%) [48].
Eckol **4**	*Ecklonia* sp. and *Eisenia* sp. [49,50]	ICR mice	75 nmol/mouse	Inhibition of ear edema induced by AA (12.7%), by TPA (40.0%), and by OXA (19.3%) [51].
		Kunming mice	0.5 mg/kg (b.w.)	Hepatoprotection by reduction of ALT (41.6%) and AST (26%) on CCl_4_-induced liver injury; decrease in expression of caspase-3 (77%), TNF-α (23%), IL-1β (%), IL-6 (26%), and lipid peroxidation (21%); increase in expression of Bcl-2 (33.3%) and IL-10 (33%). Increase in GSH (31%) and SOD (19.5%) [52].
		ICR mice	50 mg/kg (b.w.)	Anticoagulant action by increasing tail bleeding time (135%). Less active than heparin [53].
		ICR mice	20 mg/kg (b.w.)	Enhanced jejunal crypt survival (17.7%) and reduction of apoptotic cells (37.5%) in jejunal crypts after γ-ray exposure [33].
		C57BL/6 mice	10 mg/kg (b.w.)	Radioprotection increasing survival rate (58%), hematopoietic recovery (50%), reduction of DNA damage in lymphocytes (27.8%), and increase in CD3^+^ T cell (44.3%) and CD45R/B220+ pan B cell (27.6%) populations after γ-ray exposure [54].
		C57BL/6 mice	10 mg/kg (b.w.)	Inhibition of γ-radiation-induced lymphocyte apoptosis (33.33%), and intestinal cell apoptosis (16.63%) [55].
		Sprague-Dawley rats	20 mg/kg (b.w.)	Anti-hyperlipidemic effect by reduction of TG (27.2%), TC (38.6%), AI (49%), and LDL (56.5%) level and increased level of HDL (10.5%). Activity level similar to lovastatin [56].
		ICR mice	20 μM	Suppression of acetic acid-induced vessel hyperpermeability (50%) and leucocyte migration (50%) [31].
		Zebrafish	50 μM	Photoprotection by reduction of UV-B induced ROS formation (43%), NO levels (33%), cell death (78%), and hyperpigmentation (50%) [57].
Dieckol **5**	*Ecklonia* sp. and *Eisenia* sp. [49,58]	IgE/antigen-sensitized mice	20 mg/kg (b.w.) *	Administration prior to IgE sensitization, reduced mast cell degranulation, and edema formation (80%) [59].
		Sprague-Dawley rats	20 mg/kg (b.w.)	Reduction of TG (31%), TC (43.4%), AI (72.6%), and LDL (75.5%) level and increased level of HDL (35.4%). More active than lovastatin [56].
		ICR mice	20 μM	Suppression of acetic acid-induced vessel hyperpermeability (70%) and CMC-induced leucocyte migration (55%) [31].
		C57BL/KsJ-db/db mice	20 mg/kg (b.w.)	Antidiabetic effect by reduction of lipid peroxidation (87%) body weight (7%), blood glucose (40%), and blood insulin (50%). Increased the activity of SOD (8.5%), CAT (0.5%), and GSH-px (0.1%), and over-expression of AMPK (60%) and Akt (100%) [58].
		ICR mice	50 mg/kg (b.w.)	Anticoagulant effect by increasing tail bleeding time (173.8%). Less active than heparin [53].
		Zebrafish embryos	20 μM	Reduction of heart rate (13%), ROS formation (35%), NO level (18%), lipid peroxidation (10%), and cell death (10%) in high glucose-induced oxidative stress. Reduction of over-expression of iNOS (20%) and COX-2 (15%) [60].
		Zebrafish embryos	20 μM	Reduction of ROS formation (80%), lipid peroxidation (5%), and cell death (15%) on ethanol-induced damage [61].
Phlorofucofuroeckol A **6**	*Eisenia arborea* Areschouga ^a^ [51,62]; *Ecklonia stolonifera* Okamura [63]	Zebrafish embryos	41.5 μM	Decreased AAPH-induced ROS levels (40%), lipid peroxidation (48%), and cell death (70%) [64].
		ICR mice	75 nmol/mouse	Inhibition of ear edema induced by AA (30.5%), by TPA (31.7%), and by OXA (23.4%). EGCG inhibits 12.9%, 13.8%, and 5.7% of ear edema induced by AA, TPA, and OXA, respectively [51].
Phlorofucofuroeckol B **7**	*Eisenia arborea* Areschoug ^a^ [51,62]; *Ecklonia stolonifera* Okamura [63]	ICR mice	75 nmol/mouse	Inhibition of ear edema induced by AA (42.2%), by TPA (38.4%), and by OXA (41.0%). EGCG inhibits 12.9%, 13.8%, and 5.7% of ear edema induced by AA, TPA, and OXA, respectively [51].
6,6′-Bieckol **8**	*Eisenia arborea* Areschoug ^a^ [51,65]; *Ecklonia stolonifera* Okamura [63]	SHR rats	20 mg/kg (b.w.)	Reduction of 28.6 mmHg in systolic blood pressure, against 31.3 mmHg obtained with captopril [66].
		ICR mice	75 nmol/mouse	Inhibition of ear edema induced by AA (41.9%), by TPA (34.2%), and by OXA (17.8%). EGCG inhibits 12.9%, 13.8%, and 5.7% of ear edema induced by AA, TPA, and OXA, respectively [51].
6,8′-Bieckol **9**	*Eisenia arborea* Areschoug ^a^ [51,62]	ICR mice	75 nmol/mouse	Inhibition of ear edema induced by AA (39.8%), by TPA (49.4%), and by OXA (77.8%). EGCG inhibits 12.9%, 13.8%, and 5.7% of ear edema induced by AA, TPA, and OXA, respectively [51].
8,8′-Bieckol **10**	*Eisenia arborea* Areschoug ^a^ [51]	ICR mice	75 nmol/mouse	Inhibition of ear edema induced by AA (21.0%), by TPA (31.7%), and by OXA (32.3%). EGCG inhibits 12.9%, 13.8%, and 5.7% of ear edema induced by AA, TPA, and OXA, respectively [51].
Eckstolonol **11**	*Ecklonia cava* Kjellman [67], *Ecklonia stolonifera* Okamura [68]	C57BL/6N mice	50 mg/kg (b.w.)	Decrease in sleep latency and increase (1.4×) in the amount of NREMS [67].

* Unit converted for comparison purposes. ^a^ The current accepted name is *Ecklonia arborea* (Areschoug) M. D. Rothman, Mattio and J. J. Bolton.

**Table 2:** Summary of in vivo activity of seaweed peptides.

Compound	Algae	Model	Activity	Dose
Griffithsin **12**	*Griffithsia* sp. [82]	Balb/c mice	100% of mice survival from a high dose of SARS-CoV (compared to 30% survival in control group) [83].	10 mg/kg (b.w.)/day
		Balb/c mice	Protected 100% of mice from a lethal JEV dose (compared to 0% survival in control) [84].	5 mg/kg (b.w.)/day
		Chimeric uPA^+^/^+^-SCID mice	Protected mice from hepatitis C infection (viral load below detection limit in treated mice) [85].	5 mg/kg (b.w.)/day
		Balb/c mice	Significantly protected mice from HSV-2 vaginal infection (0/5 treated mice were infected compared to 3/5 infected in control group, after 7 days) [86].	20µL of 0.1% griffithsin gel
		New Zealand rabbits	Caused no mucosal damage or inflammatory responses with intravaginal administration [87].	0.1% griffithsin gel
		Balb/c mice	Significantly protected mice from HSV-2 vaginal infection and HPV16 pseudovirus challenge [88].	20 µL gel of griffithsin–carragenan combination (0.1% **12** and 3% CG)
		Rhesus macaques	Did not negatively impact the mucosal proteome or microbiome [89].	0.1% griffithsin gel
Tridecapeptide **13**	*Palmaria palmata* (Linnaeus) F. Weber and D. Mohr [90]	SHR mice	After 2 h, significant 33 mmHg SBP reduction; captopril at same dose caused 29 mmHg SBP reduction [90].	3 mg/kg (b.w.)
Dipeptide **14**	*Undaria pinnatifida* (Harvey) Suringar [91]	SHR mice	16 mmHg SBP reduction after 3 h; captopril at same dose caused 17 mmHg SBP reduction [91].	1 mg/kg (b.w.)
Phycoerythrin **15**	*Porphyra haitanensis* T.J. Chang and B.F. Zheng ^a^*, Grateloupia turuturu* Yamada, *Gracilaria lemaneiformis* (Bory) Greville ^b^ [92,93,94]	S180 tumor-bearing mice	Reduced tumor growth by 41.3%. Increase TNF-α level, lymphocyte proliferation, and SOD activity [92].	300 mg/kg (b.w.)
		N2 *Caenorhabditis elegans*	Increased *Caenorhabitis elegans* lifespan (15 ± 0.1 to 19.9 ± 0.3 days), increased thermal stress resistance (22.2% ± 2.5% to 41.6% ± 2.5% mean survival) and oxidative stress resistance (30.1% ± 3.2% to 63.1% ± 6.4% mean survival) [95].	100 µg/mL
		CL4176 *Caenorhabitis elegans*	Significant reduction of senile plaque formation (2-fold reduction in grayscale values [96].	100 μg/mL
Kahalalide F **16**	*Bryopsis* sp. [97]	Athymic mice with xenografted tumors	Reduced prostate tumor growth by 50% and 35% [98].	0.245 and 0.123 mg/kg (b.w.)

^a^ The current accepted name is *Pyropia haitanensis* (T. J. Chang and B. F. Zheng) N. Kikuchi and M. Miyata). ^b^ The current accepted name is *Gracilariopsis lemaneiformis* (Bory de Saint-Vincent) E. Y. Dawson, Acleto and Foldvik.

**Table 3:** Summary of in vivo activity of halogenated terpenoids and bromophenols seaweed compounds.

Compound	Source	Model	Activity	Dose
Halomon **17**	*Portieria hornemanii* (Lyngbye) P.C. Silva [114]	U251 brain tumor ip/ip xenograft mouse model	40% “apparent cures” of mouse brain cancer [114].	5 × 50 mg/kg (b.w.)
Neorogioltriol **18**	*Laurencia glandulifera* (Kützing) Kützing [115]	Swiss mice and rats	Reduce writhing response by 88.9% and reduced pain response behavior by 48% [115].	1 mg/kg (b.w.)
		Rats	Reduced paw swelling by 58% after 3 h. 300 mg/kg (b.w.) of acetylsalicylic acid was required to obtain the same effect [116].	1 mg/kg (b.w.)
Neorogioldiol **19**	*Laurencia glandulifera* (Kützing) Kützing, *Laurencia microcladia* Kützing [117,118]	C57BL/6 mice	Reduced inflammatory colon damage and cytokine expression (reduced IL-1β by 6-fold and IL-6 by 40-fold) [117].	0.25 mg/kg (b.w.)
*O*^11^,15-cyclo-14-bromo-14,15-dihydrorogiol-3,11-diol **20**	*Laurencia glandulífera* (Kützing) Kützing [117]	C57BL/6 mice	Reduced inflammatory colon damage and cytokine expression (reduced IL-1β by 7-fold and IL-6 by 40-fold) [117].	0.25 mg/kg (b.w.)
BDDE **21**	*Odonthalia corymbifera* (S.G. Gmelin) Greville [119], *Leathesia nana* Setchell and N.L. Gardner ^a^ [120], *Rhodomela confervoides* (Hudson) P.C. Silva [121].	Zebrafish embryos	Reduced SIV growth by 17.7%, 40.4%, and 49.5% [121].	6.25, 12.5, and 25 mM
		Db/db mice	Reduction of blood glucose levels (12.3%) (metformin caused a 10.1% decrease). Decreased glycated hemoglobin, triglycerides and body weight [122].	40 mg/kg (b.w.)

^a^ The current accepted name is *Leathesia marina* (Lyngbye) Decaisne.

## Abbreviations

AA	Arachidonic acid
AAPH	2.2′-azobis (2-amidinopropane)
ACE	Angiotensin-converting-enzyme
AI	Atherogenic index
ALI	Acute lung injury
AMPK	Adenosine monophosphate-activated protein kinase
AOM	Azoxymethane
AP-1	Activator protein-1
BALB/c	Strain of laboratory mouse
BALF	Broncho-alveolar lavage fluid
Bax	Bcl-2-associated X
Bcl-2	B-cell lymphoma 2
BDDE	Bis(2,3-dibromo-4,5-dihydroxybenzyl) ether
BDNF	Brain-derived neurotrophic factor
BMI	Body mass index
b.w.	Body weight
C57BL/6	Strain of laboratory mouse
C57BL/6J	Strain of laboratory mouse
C57BL/KsJ-db/db	Strain of laboratory diabetic mouse
CAT	Catalase
CD_2_F_1_	Strain of laboratory mouse
CD4	Cluster of differentiation 4 cells
CG	Carragenan
CMC-	Carboxy-methylcellulose
COX-2	Cyclooxygenase-2
CTx	C-terminal telopeptide of type-1 collagen
DNA	Deoxyribonucleic acid
DSS	Dextran sodium sulfate
DU-145	Human prostate cancer cell line
E2	Estradiol
EC_50_	Half maximal effective concentration
EGCG	Epigallocatechin gallate
ER	Endoplasmic reticulum
ERCC1	Excision repair cross-complementation
FD	Fine dust
GABAA-BZD	Gamma-aminobutyric acid A-benzodiazepine
GRP78	Glucose-regulated protein 78
GSH-px	Glutathione peroxidase
HDL	High-density lipoprotein
HIV	Human immunodeficiency virus
HPV16	Human papillomavirus type 16
HSV-2	Herpes simplex virus type 2
IC_50_	Half maximal inhibitory concentration
ICR	Strain of laboratory mouse
IgE	Immunoglobulin E
IL-1	Interleukin-1
IL-6	Interleukin-6
IL-10	Interleukin-10
iNOS	Inducible nitric oxide synthase
IU	International unit
JEV	Japanese encephalitis virus
JNK	c-Jun NH2-terminal kinase
Ki-67	Proliferation marker protein
LDL	Low-density lipoprotein
LPS	Lipopolysaccharides
MAPK	Mitogen-activated protein kinase
MCAO	Middle cerebral artery occlusion rat model
MDA-MB-231	Human breast adenocarcinoma
MKK4/SEK1	Mitogen-activated protein kinase kinase-4
MNZ	Metronidazole
mRNA	Messenger ribonucleic acid
NCI	National Cancer Institute
NER	Nucleotide excision repair
NF-_K_B	Nuclear factor kappa B
NK	Natural killer cells
NO	Nitric oxide
NREMS	Non-rapid eye movements
OXA	Oxazolone
PC-3	Human prostate cancer cell line
PM2.5	Particulate matter ≤2.5 μm
PPARγ	Peroxisome proliferator-activated receptor gamma
ROS	Reactive oxygen species
S180	Murine sarcoma cancer cell line
sAβ1-42	Soluble amyloid beta peptide (1-42)
SAR	Structure–activity relationship
SARS-CoV	Severe acute respiratory syndrome-related coronavirus
SBP	Systolic blood pressure
SD	Sprague-Dawley rats
SHR	Spontaneously hypertensive rats
SIV	Sub-intestinal vessel
SOD	Superoxide dismutase
TC	Total cholesterol
TG	Triglycerides
TNF	Tumor necrosis factor
TNF-α	Tumor necrosis factor α
TPA	12-*O*-tetradecanoylphorbol-13-acetate
U251	Human glioblastoma
uPA-SCID	Urokinase-type plasminogen activator severe combined immunodeficient mice
UV	Ultraviolet
VEGFR-2	Vascular endothelial growth factor receptor 2
XPC	Xeroderma pigmentosum complementation group C
